# Molecular epidemiology of antimicrobial resistance in central africa: A systematic review

**DOI:** 10.1099/acmi.0.000556.v5

**Published:** 2023-08-16

**Authors:** Annicet-Clotaire Dikoumba, Richard Onanga, Laurette G. Mangouka, Larson Boundenga, Edgard-Brice Ngoungou, Sylvain Godreuil

**Affiliations:** ^1^​ Département de biologie médicale, Hôpital d’Instruction des Armées Omar Bongo Ondimba, B.P 20404 Libreville, Gabon; ^2^​ Unité de recherche et d’Analyses Médicales (URAM), Centre Interdisciplinaire de Recherches Médicales de Franceville (CIRMF), B.P. 679 Franceville, Gabon; ^3^​ Département de Médecine, Hôpital d’Instruction des Armées Omar Bongo Ondimba, B.P 20404 Libreville, Gabon; ^4^​ Groupe Evolution et Transmission Inter-espèces des Pathogènes, Département de Parasitologie du Centre Interdisciplinaire de Recherches Médicales de Franceville (CIRMF), Franceville, Gabon; ^5^​ Unité Maladies Émergentes Virales, Département de Virologie du Centre Interdisciplinaire de Recherches Médicales de Franceville (CIRMF), Franceville, Gabon; ^6^​ Unité de Recherche en Epidémiologie des Maladies Chroniques et Santé Environnement (UREMCSE), Département d’Epidémiologie, Biostatistiques et Informatique Médicale (DEBIM), Faculté de Médecine, Université des Sciences de la Santé, BP 4009 Libreville, Gabon; ^7^​ Laboratoire de Bactériologie, Centre Hospitalier Universitaire de Montpellier, 191 Avenue du Doyen Gaston Giraud, 34 295 Montpellier Cedex 5, France; ^8^​ MIVEGEC, IRD, CNRS, Université de Montpellier, Montpellier, France

**Keywords:** resistance genes, genetic carriers, Central Africa, One Health

## Abstract

**Background.:**

In Central Africa, it is difficult to tackle antibiotic resistance, because of a lack of data and information on bacterial resistance, due to the low number of studies carried out in the field. To fill this gap, we carried out a systematic review of the various studies, and devised a molecular epidemiology of antimicrobial resistance from humans, animals and the environmental samples.

**Method.:**

A systematic search of all publications from 2005 to 2020 on bacterial resistance in Central Africa (Gabon, Cameroon, Democratic Republic of Congo, Central African Republic, Chad, Republic of Congo, Equatorial Guinea, São Tomé and Príncipe, Angola) was performed on Pubmed, Google scholar and African Journals Online (AJOL). All circulating resistance genes, prevalence and genetic carriers of these resistances were collected. The study area was limited to the nine countries of Central Africa.

**Results.:**

A total of 517 studies were identified through a literature search, and 60 studies carried out in eight countries were included. Among all articles included, 43 articles were from humans. Our study revealed not only the circulation of beta-lactamase and carbapenemase genes, but also several other types of resistance genes. To finish, we noticed that some studies reported mobile genetic elements such as integrons, transposons, and plasmids.

**Conclusion.:**

The scarcity of data poses difficulties in the implementation of effective strategies against antibiotic resistance, which requires a health policy in a ‘One Health’ approach.

## Data Summary

No data was reused or generated.

## Introduction

Antimicrobial resistance is currently considered an emerging global threat and a major public health problem [1]. The epidemiology of antibiotic resistance among clinical pathogens is essential, particularly for therapeutic management [2]. However, limited data are available in Central Africa [3]. Currently, the main multi-resistant bacteria (MRB) responsible for infectious diseases are methicillin-resistant *Staphylococcus aureus* (MRSA), extended-spectrum beta-lactamase-producing Enterobacteriaceae (ESBL-PE) or cephalosporinase-producing Enterobacteriaceae, ceftazidime-resistant *Pseudomonas aeruginosa* (CRPA), ESBL-producing *Acinetobacter baumanii*, vancomycin-resistant enterococci (VRE), and carbapenemase-producing Gram-negative bacilli (Enterobacteriaceae, *Pseudomonas aeruginosa *, *Acinetobacter baumanii*) [4].

It should be noted that ESBL-PE are now the most predominant MRBs in many countries [5]. They frequently have resistance to other antibiotics such as fluoroquinolones and aminoglycosides [6]. Carbapenemases are an heterogeneous group of enzymes whose spectrum of activity covers at least one of the carbapenems. In Africa, this type of resistance remains scarce but is related to the risk of therapeutic impasse. Indeed, carbapenems have often been used as the treatment of choice for infectious diseases due to ESBL-PE. This has led to the emergence of carbapenemase-producing Enterobacteriaceae (CPE), which are enzymes hydrolysing all beta-lactams, even carbapenems which are the last line in the treatment for ESBL-PE [7, 8]. There are limited data assessing the impact of different factors on the current rate of antimicrobial resistance in low-income countries [9–11].

While reliable data exist in Europe, the United States, Latin America [12] and Asia [13, 14], very little is available in sub-Saharan Africa [3], particularly in Central Africa where, despite efforts, research on antibiotic resistance is still very weak.

There are two factors in the development of antibiotic resistance, both interdependent and linked to human activity: the excessive use of antibiotics in human and animal health, which leads to the selection of the most resistant bacteria, and the spread of the selected resistant bacteria, through direct transmission within humans and animals (‘cross-transmission’), and indirectly through the environment [15]. Horizontal gene transfer (HGT) contributes significantly to the rapid spread of resistance. Multiple mechanisms of HGT liberate genes from normal vertical inheritance. Conjugation by plasmids, transduction by bacteriophages, and natural transformation by extracellular DNA each allow genetic material to jump between strains and species. Thus, HGT adds an important dimension to infectious disease whereby an antibiotic resistance gene can be the agent of an outbreak by transferring resistance to multiple unrelated pathogens [16]. Thus, the use of antibiotics in veterinary medicine and the discharge of antibiotics into the environment contribute to the emergence of new multidrug-resistant bacterial strains. To combat antibiotic resistance, it is necessary to follow a ‘One Health’ approach that joins efforts of the human, animal and environmental health compartments [17].

In Africa, antimicrobial resistance is increasing to worrisome proportions. Salah *et al*. demonstrate, between 2010 and 2017, an increase in antibiotic resistance of Enterobacteriaceae isolated at the National Institute of Hygiene of Lomé. During this period, the rate of resistance to ceftazidime of *Escherichia coli* strains increased significantly from 18.69–39.26 % (*P*<0.0001); from 1.68–40.22 % to ceftriaxone (*P*<0.0001) and from 42.37–63.23 % (*P*<0.0001) to ciprofloxacin. Resistance of *Klebsiella* spp. strains to ceftazidime increased significantly from 25.26–42.54 % (*P*<0.0001) and to ceftriaxone from 2.17–41.79 % (*P*<0.0001) [18]. It is therefore becoming imperative, in order to combat effectively against the emergence of these resistances, to control the favouring factors and the epidemiology of the different circulating resistance genes and their genetic supports. In this study, we carried out a systematic review of the literature, to collect and compile available data on bacterial resistance in Central Africa, in order to map the circulating resistance genes and their carriers in humans, animals and the environment.

## Bibliographical methods

### Study strategy

For this systematic review, we used methodology suggested by the Preferred Reporting Items for Systematic Reviews and meta-analyses as described [19]. Thus, we performed a systematic search of databases such as Medline (Pubmed), Google scholar and African Journals Online (AJOL). Our research focused on the collection of research articles related to antimicrobial resistance in Central Africa published between January 2005 and September 2020. In order to ensure a full search, including all available studies on the topic and in the study area, we have used a combination of keywords. Search terms were ‘antibiotic resistance genes’, or ‘ESBLs’, or ‘antimicrobial drug resistance’ associated with the name of a study area country (Central Africa) as search strategies. The aim was to identify all the circulating resistance genes in the Sub-Saharan region, their prevalence rates and genetic supports (plasmid or chromosome).

### Study area

**Fig. 1.**
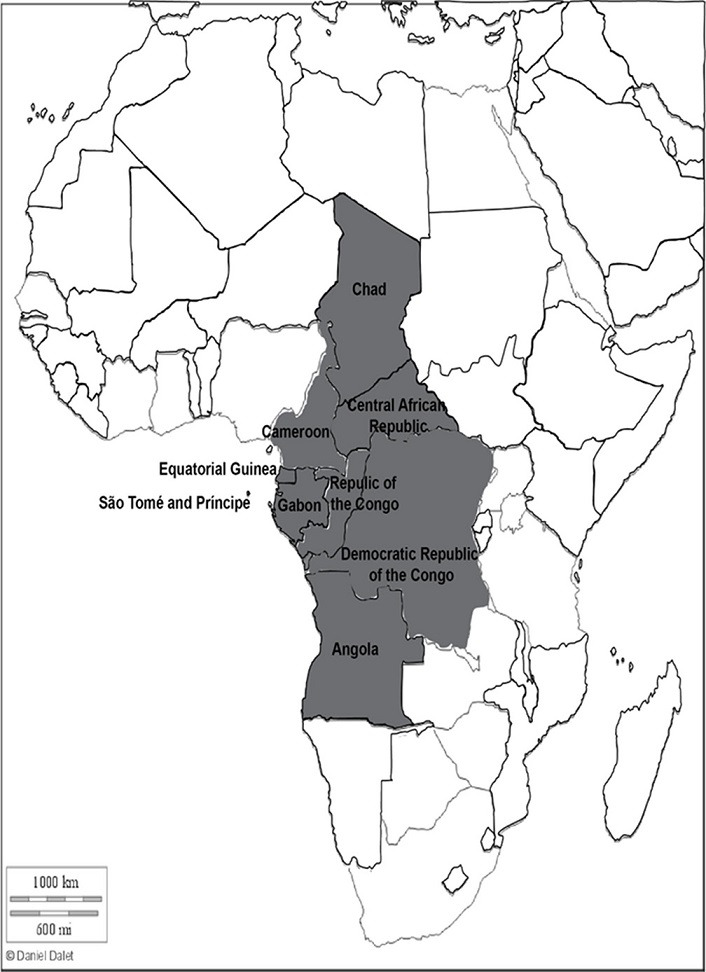
African card 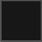
Study area (Central Africa)

Our study concerns Central Africa, and this Sub-Saharan region of Africa according to the United Nations is made up of nine countries including Gabon, Cameroon, Democratic Republic of Congo (DRC), Central African Republic (CAR), Chad, Republic of Congo, Equatorial Guinea, São Tomé and Príncipe, Angola (Fig. 1). The Central Africa region covers an overall area of 6 613 000 km^2^ and in 2017, the population was estimated around 163 495 000. In this area, the mean population density of 25 inhabitants per km^2^ [20].

### Criteria for inclusion in the review and for ineligibility

All studies on bacterial resistance (in English or French), in humans, animals and the environment, which were published within January 2005 to September 2020 were selected. We therefore included studies whose methodology allowed the identification of resistance genes and/or genetic supports.

We did not include studies carried out in countries outside the study area (Central Africa), and studies that reported only the results of antibiotic susceptibility testing or antimicrobial resistance monitoring, with no molecular tests to characterize resistance genes and/or genetic supports.

### Data extraction

After removing studies not fulfilling the eligibility criteria and the duplicates, and after a full reading of each selected article, an essential database for the review was created, including: country, sample source, resistance genes found, prevalence rate of resistance, genetic support for resistance genes, and year of the study, (Tables 1–3) referring to studies in humans, animals and the environment, respectively.

**Table 1.** Data on bacterial resistance in humans in Central Africa

Country	no. of reports	Source of samples	ESBL and other β-lactamases genes	Rate of ESBL-PE (%)	Carbapenemase genes	Rate of CPE (%)	Other resistance genes	Rate of other resistance genes (%)	Genetic elements	Study period	References
**Gabon**	11	Clinic and	*CTX-M, CTX-M15,*	3–100	*NDM-7*	5.1	**Methicillin**	0.97–96	Transposon	2012 (*n*=1)	[43–45]
		carriage	*CTX-M1, CTX-M8,*		*OXA-48*		*mecA*		IncX3	2013 (*n*=3)	[46–53]
			*CTX-M27, TEM,*				**Aminoglycosides**		Integrons classes 1 et 2 (IntI1, IntI2)	2014 (*n*=4)	
			*TEM-104, SHV,*				*aac(6')-ib, aac(3)-IIa*		SCCmec IV	2016 (*n*=3)	
			*SHV-12, SHV-28,*				*ant(3), aac(3)*		SCCmec V		
			*OXA, OXA-9,*				*aadA1, aphA-3*		SCCmec non-typable		
			*OXA-30, blaZ,*				**Sulfonamides**				
			*act-17, ampR,*				*sul1, sul2, folA*				
			*lap-2*				**Trimethoprin**				
							*dhfr 1, dfrA,*				
							*dfrG, dfrK +G, dfrB*				
							***Tetracyclines***				
							*tetA-2, tetD,*				
							*tetK, tetM*				
							**Quinolones**				
							*qnrS1*				
							**Fosfomycin**				
							*fosA*				
							**Rifampicin**				
							*arr-ms*				
							**Macrolides**				
							*mphA,*				
							*mpbBM, msr(A*)				
							**Chloramphenicol**				
							*cat*				
**Angola**	8	Clinic and	*CTX-M15, CTX-M55,*	22.2–28	*OXA-181,*	27.4 à 78	**Methicillin**	2.5–56	IncX3, IncFIA,	2006 (*n*=1)	[54–56]
		carriage	*TEM-1,*		*NDM-1,*		*mecA*		IncA/C,	2011 (*n*=1)	[57–61]
			*OXA-1, blaP1*		*NDM-5*		**Aminoglycosides**		IncFIB, IncL/M, IncN,	2015 (*n*=1)	
							*armA, rmtB*		IncY, IncFII, IncHI2	2016 (*n*=2)	
							*rmtC, aadA1*		Integrons classes		
							*aadA8, aph*		one et 2 (int1 and int2)		
							**Streptomycin**		Plasmide non-typable		
							strA, strB		p3iANG		
							**Quinolones**		SCCmec Iva		
							*aac(6')-ib-cr,*		SCCmec V		
							*qnrB, qnrS,*				
							**Trimethoprin**				
							*dfrA1, dfrA15,*				
							*dfrA18*				
							**Sulfonamides**				
							*sul1, sul2*				
							**Tetracyclines**				
							*tetG*				
							**Chloramphenicol**				
							*cat1, floR*				
**Cameroon**	11	Clinic and	*CTX-M15, OXA-1,*	2–96	*NDM-1, NDM-4*		**Aminoglycosides**	1.79–85.71	pYC-5b, pYC-14	2005 (*n*=2)	[62–64]
		carriage	*OXA-9, OXA-30,*				*aacA4, aac(3)-Iia,*		IncFIA, CoIRNAI,	2012 (*n*=3)	[65–67]
			*OXA-50, OXA-395,*				*aadA1, aac(6')-Ib,*		IncFIB(K), IncFIA(HI1),	2016 (*n*=1)	[68–70]
			*OXA-486, TEM-1,*				*aac(3)-Iid, aadA16*		IncHI1B, IncR, CoI,	2018 (*n*=2)	[71, 72]
			*TEM_1A, TEM-1B,*				*aph(3')-IIb, aph(3')-Ib,*		IncY,	2020 (*n*=3)	
			*TEM-116, SHV-1,*				*aph(6')-Id, rmtB*		Integron class 1 (int 1)		
			*SHV-11, SHV-12,*				**Streptomycin**		Transposons		
			*SHV-134, PSE-1,*				*strA, strB*		(Tn4651, Tn4652)		
			*PAO, SCO-1*				**Quinolones**				
							*oqxA, oqxB,*				
							*QnrB1, aac(6')-Ib-cr,*				
							*crpP, gyrA, parC,*				
							*parE, QnrS1*				
							**Sulfonamides**				
							*sul1, sul2, sul3*				
							**Trimethoprim**				
							*dfrA15, dfrA27,*				
							*dfr12, dfr7,*				
							*dfr1a, dfrA14*				
							*dfrA1*				
							**Fosfomycin**				
							*fosA*				
							**Tetracyclines**				
							*tetA, tetB, tetD, tetG, tetR*				
							**Chloramphenicol**				
							*catA2, cat1,*				
							*cat2, catB7,*				
							*catB9, cmIA, floR*				
							**Rifampicyn**				
							*ARR-3*				
							**macrolides**				
							*mph(A*)				
							**Glycopeptides**				
							*vanW*				
											
**Chad**	3	Clinic and	*CTX-M15, CTX-M27,*	48	*NDM-5*	2.5–6.5	**Aminoglycosides**	0.97–94	IncX3, IncF, IncR	2019 (*n*=3)	[73–75]
		carriage	*CTX-M9, CTX-M14,*		*OXA-181*		*armA, rmtB, rmtC*		non-typeable plasmid,		
			*OXA-1, TEM-1*				**Quinolones**		Mobp 5–3 plasmid		
							*qnrS, qnrB, qnrD,*				
							*oqxAB, aac(6')-Ib-cr,*				
							*qepA*				
											
**Democratic**	6	Clinic and	*CTX-M15, SHV-12,*	10	na	–	**Aminoglycosides**	–	IncFIB69, IncFII105,	2012 (*n*=1)	[76–78]
**Republic of Congo**		carriage	*OXA-1, TEM-1*				*AAC3, ANT2,*		IncFII107,	2013 (*n*=1)	[79–81]
			*SHV-2a, TEM-1b,*				*ANT3, APH3, APH6,*		SCCmecV	2015 (*n*=2)	
			*CMY*				*aac(6'), aph(2''*)			2017 (*n*=1)	
							*aph(3'), ant(4'),*			2019 (*n*=1)	
							*aac(6')-Iaa*				
							**Quinolones**				
							*aac(6')-Ib-cr, qnrB,*				
							*qnrB1, qnrS, gyrA*				
							**Tetracyclines**				
							*tetA, tetB,*				
							*tetD, tetM, tetK*				
							**Sulfonamides**				
							*folP, sul1, sul2*				
							**Macrolides**				
							*mphA, erm(A),*				
							*erm(B), erm, erm(T*)				
							**Chloramphenicol**				
							*cat, catA, catB, catA1*				
							**Rifampicyn**				
							*Arr*				
							**Trimethoprim**				
							*DHFR, dfrG,*				
							*dfrK, dfrA1*				
							**Methicillin**				
							*mecA*				
							**Streptomycin**				
							*strA, strB*				
											
**Republic of**	3	clinic	*CTX-M15, TEM-1,*	9.30–74.42	*OXA-181*	6.97	**Methicillin**	22.22–60	na	2019 (*n*=3)	[82–84]
**Congo**			*SHV-1, SHV-85*				*mecA*				
							**Colistin**				
							*mcr-1*				
**Central**	6	Clinic and	*CTX-M15, CTX-M3,*	–	na	–	**Aminoglycosides**	13–97	IncF, IncHI2,	2006 (*n*=1)	[85–87]
**African** **Republic**		carriage	*CTX-M27, CTX-M127*				*aac(6')-Ib, aacC3*,		IncFIB, IncQ1	2007 (*n*=1)	[88–90]
			*TEM-1, OXA-1,* *OXA30,*				*aadA1, aadA2,aadA5*			2014 (*n*=1)	
			*SHV-2a, SHV-12*				*tmrB*			2015 (*n*=1)	
							**Streptomycin**			2016 (*n*=1)	
							*strA, strB*			2019 (*n*=1)	
							**Chloramphenicol**				
							*catB3, catA1*				
							**Trimethoprim**				
							*dfrA14, dfrA7,*				
							*dfrA1, dfrA5, dfrA2d*				
							*dfr12, dfrA17*				
							**Sulfonamides**				
							*sul1, sul2, sul3*				
							**Tetracyclines**				
							*tetA, tetB, tetD*				
							**Quinolones**				
							*aac(6')-Ib-cr, qnrB,*				
							*qnrB1, qnrS, oqxA,*				
							*oqxB, qepA, qnrS1*				
**São Tome and**	2	carriage	*CTX-M15, TEM-1*	–	*Oxa-181*	44	**Methicillin**	2.78–16	SCCmec Iva	2018 (*n*=2)	[59, 91]
**Príncipe**							*mecA*		IncX3		
							**Aminoglycosides**		IncX4		
							*rmtB*				
							**Colistin**				
							*mcr-1*				

ESBL: Extended Spectrum Beta-lactamase; ESBL-PE: Extended Spectrum Beta-lactamase-Producing Enterobacteriaceae; CPE: Carbapenemase-Producing Enterobacteriaceae; %: Percentage

N.B: One study, carried out in both countries, was recorded in Angola and Sao Tome and Principe.

**Table 2.** Data on bacterial resistance in animals in Central Africa

Country	Number of reports	Source of samples	ESBL and other β-lactamases genes	Rate of ESBL-PE (%)	Carbapenemase genes	Rate of CPE (%)	Other resistance genes	Rate of other resistance genes (%)	Genetic elements	Study period	References
Gabon	3	Gorilla	*CTX-M1, CTX-M14,*	5.9–41.2	na	–	**Aminoglycosides**	50	na	2014 (*n*=1)	[49, 92]
		Chicken	*CTX-M15, CTX-M32*				*aac(6')-ib, acc(3)-II*			2015 (*n*=1)	[93]
		Bat	*TEM, SHV, SHV-11*				*aadA1, aadA2,*			2020 (*n*=1)	
							*aadA5*				
Angola	2	Cow	*CTX-M15*	25–75	na	–	**Streptomycin**	56	IncFIB, IncY,	2014 (*n*=1)	[60, 94]
		Pig	*TEM-1*				*strA*		IncN, IncI1,	2015 (*n*=1)	
		Chicken	*OXA-1*				**Quinolones**		IncFIIk6,		
							*aac(6')-ib-cr, qepA,*		IncFII36		
							*qnrB, qnrS*				
							**Tetracyclines**		Integrons classes		
							*tetA, tetB, tetD*		one et 2 (int1,int2)		
							**Sulfonamides**				
							*sul1, sul2*				
							**Trimethoprin**				
							*dfrA15*				
							**Chloramphenicols**				
							*catA1, cmlA*				
Cameroon	2	Pig	, *CTX-M15*	21.52	na	–	**Methicillin**	–	SCCmectypeVc	2018 (*n*=2)	[67, 95]
			*TEM-116, SHV-1, TEM-1B,*				*mecA*		CoIRNAI, CoIE10,		
			*SHV-27, SHV-28,*				**Macrolides**		IncFIB (K),		
			*blaZ, SCO-1*				*ermB, ermC,*		IncFIA(HI1),		
							*mph(A)*		IncY, IncFII(K), IncR, CoIE10		
							**Tetracyclines**				
							*tetA, tetK,*				
							*tetL, tetM*				
							**Aminoglycosides**				
							*aac(3')-IIa, aadA1*				
							**Streptomycin**				
							*strA, strB*				
							**Quinolones**				
							*oqxA, oqxB,*				
							*QnrB1*				
							**Fosfomycin**				
							*fosA*				
							**Sulfonamides**				
							*sul1, sul2*				
							**Trimethoprim**				
							*dfrA15*				
							**Chloramphenicols**				
							*catA1*				
Central	1	gorillas,		–	na	–	**Aminoglycosides**	–	na	2014	[87]
African		Agile mangabeys	*CTX-M2, CTX-M15, TEM-1, SHV-62*				*aadA1, aadA2,*				
Republic		chimpanzees,					*aadA5*				
		African buffalos,					**Quinolones**				
		Forest elephants,					*qnrB33, qnrB17,*				
		Red River hogs,					*qnrB28, oqxA,*				
		duikers,					*qepA, qnrS1*				
		Lowland bongos,					**Sulfonamides**				
		Sitatunga					*sul1, sul2*				
							**Tetracyclines**				
							*tetA, tetB*				
							**Chloramphenicols**				
							*catA1*				
							**Trimethoprim**				
							*dfr12, dfrA17,*				
							*dfrA7*				
							**Streptomycin**				
							*strA, strB*				

ESBL: Extended Spectrum Beta-lactamase; ESBL-PE: Extended Spectrum Beta-lactamase-Producing Enterobacteriaceae; CPE: Carbapenemase-Producing Enterobacteriaceae; %: Percentage

**Table 3.** Data on bacterial resistance in the environment in Central Africa

Country	Number of reports	Source of samples	ESBL and other β-lactamases genes	Rate of ESBL-PE (%)	Carbapenemasegenes	Rate of CPE (%)	Other resistancegenes	Rate of other resistances (%)	Genetic elements	Studyperiod	References
											
Angola	1	Floors, walls	*CTX-M15*	30	na	–	**Quinolones**	–	IncFII36	2015 (*n*=1)	[60]
		wastewater	*TEM-1*				*aac(6')-ib-cr*		IncHI2		
		treated water for	*OXA-1*						IncY		
		humanconsumption							chromosome		
		water for							(CTX-M15)		
		animalconsumption									
		urbansewer line									
		river									
											
Cameroon	1	wastewater	*CARB3,*	5.2–15	AIM1	–	**Aminoglycosides**	2.8–80	CoIE,IncA/C,	2019 (*n*=1)	[96]
			*CARB5,*		*IMP11, IMP12*		*aac(6')−29 a, aac(6')Iia,*		IncB/O/K/Z.		
			*CfxA6, CMY1,*				*aad(6), aadA,*				
			*CMY59, GES21,*				*aac(6')-Ib7, aadA13,*				
			*NPS, AER1,*				*aadA15, aadA16,*		Inc FIA, IncFIB,		
			*OXA1, OXA164,*				*aac(6')-Ie-aph(2'')-Ia,*		IncFIC, IncFII,		
			*OXA212, OXA226,*				*aadA4, aadA7,*		IncH, IncH,		
			*OXA232, OXA256,*				*ant(2'')-Ia, ant(3')-Ii,*		IncI, IncI,		
			*OXA347, OXA-46,*				*aac(6')-Iid, ant(4')Ib,*		IncN, IncP,		
			*OXA-5, OXA-9,*				*ant(4')-Iib, ant(6)Ia,*		IncB, IncR,		
			*SHV100, TEM126,*				*ant(6)-Ib, ant(9)-Ia,*		IncT, IncU,		
			*VEB-3, LCR1*				*aph(3')-Ia, aph(3'')Ib,*		IncY, IncW,		
							*aph(3'')-Ic, aph(3')Iic,*		IncX		
							*aph(3')-IIIa, aph(6)Id,*				
							*armA*				
							**Chloramphenicol**				
							*Cat, catB3,*				
							*catI, catQ,*				
							*cat-TC, flor*				
							**Rifampicyn**				
							*ARR-3*				
							**Quinolones**				
							*oqxA, oqxB,*				
							*qnrB41, qnrD2,*				
							*qnrS6, qnrVC1,*				
							*qnrVC4*				
							**Macrolides**				
							*ereA2, ereB,*				
							*erm(33), erm(47),*				
							*ermB, ermC,*				
							*ermF, ermG,*				
							*ermQ, ermT,*				
							*ermX, lnuA,*				
							*lnuB, lnuC,*				
							*mefA, mefB,*				
							*mefCmphA,*				
							*mphE, mphG*				
							**Phosphonicantibiotics**				
							*fosA2, fosA5,*				
							*fosB*				
							**Streptogramins**				
							*vgaC*				
							**Sulfonamides**				
							*sul1, sul2, sul3*				
							**Trimethoprin**				
							*dfrA1, dfrA10,*				
							*dfrA12, dfrA14,*				
							*dfrA16, dfrA19,*				
							*dfrA23, dfrA3,*				
							*dfrA8, dfrB3,*				
							*dfrC, dfrD,*				
							*dfrE, dfrF,*				
							*dfrG*				
							**Tetracyclines**				
							*tet31, tet32,*				
							*tet33, tet36,*				
							*tet38, tet39,*				
							*tet40, tet44,*				
							*tetA(P), tetB(P),*				
							*tetC, tetD,*				
							*tetE, tetG,*				
							*tetH, tetK,*				
							*tetL, tetM,*				
							*tetO, tetQ,*				
							*tetS, tetT,*				
							*tetV, tetW,*				
							*tetX, tetY,*				
							*tetZ*				
							**Nucleosideantibiotics**				
							*sat-1, sat-4*				
Democratic	6	Wastewater +	*CTX-M1* group,	5.3–7.4	*OXA-48, KPC,*	–	**Aminoglycosides**	–	na	2012 (*n*=2)	[97, 98]
Republic of Congo		drinking water	*CTX-M, TEM,*		*VIM, IMP,*		*aadA*			2016 (*n*=1)	[99, 100]
			*SHV-2, SHV-18*		*NDM*		**Sulfonamides**			2019 (*n*=1)	[101, 102]
			*SHV, OXA,*				*sul1, sul2, sul3*			2020 (*n*=2)	
							**Tetracyclines**				
							*tetB*				
Republic of Congo	2	householdwastewater	*TEM-1*	9.30–74.42	na	–	**Methicillin**	47.36	na	2019 (*n*=2)	[82, 84]
			*CTX-M15, SHV-1, SHV-85*				*mecA*				

ESBL: Extended Spectrum Beta-lactamase; ESBL-PE: Extended Spectrum Beta-lactamase-Producing Enterobacteriaceae; CPE: Carbapenemase-Producing Enterobacteriaceae; %: Percentage

## Distribution and characteristics of the included studies

**Fig. 2.**
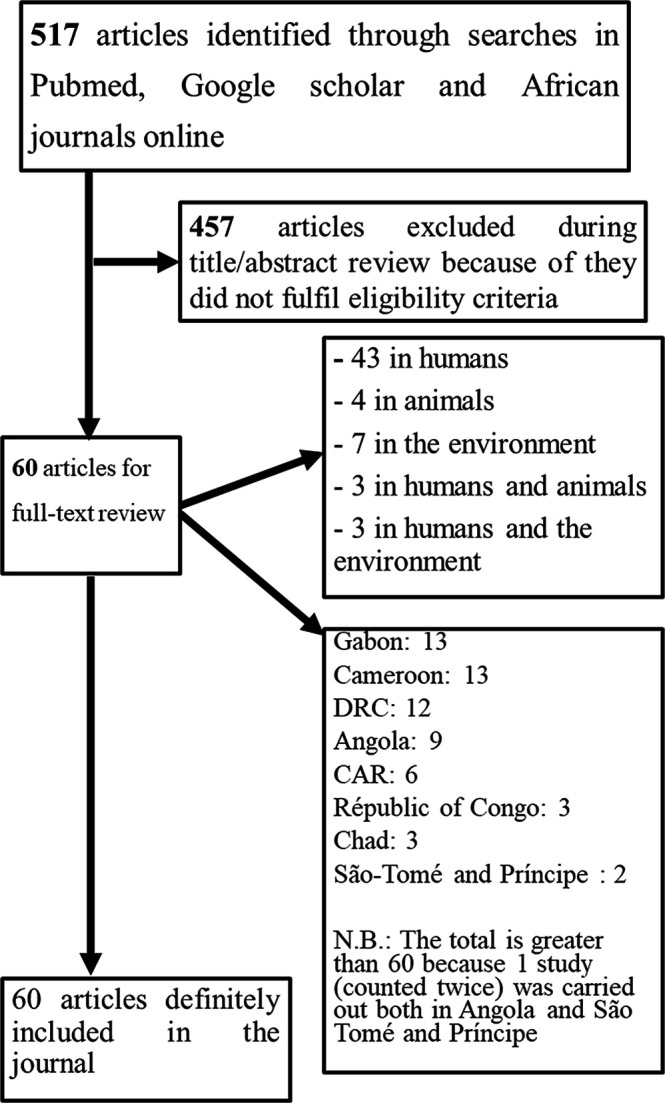
Flow chart of article selection process

A total of 517 articles were identified through the search. After reviewing each abstract, 457 articles that did not fulfil eligibility criteria were excluded. The remaining 60 articles were fully and thoroughly reviewed to extract data on bacterial resistance in Central Africa (Fig. 2). The selected articles concerned eight of the nine countries in the region.

With 13 articles each, Gabon and Cameroon had the most, followed by DRC (12), Angola (9), CAR (6), Chad and the Republic of Congo (three each), and São Tomé and Príncipe (2). It should be noted that one paper is counted twice, as the study was carried out in both Angola and São-Tomé and Príncipe. No studies fulfilling the eligibility criteria were found in Equatorial Guinea.

Among the 60 included studies, 43 were carried out in humans, four in animals, seven in the environment, three in humans and animals, three in humans and the environment.

An antimicrobial susceptibility testing was performed in 48 studies, of which 38 used the agar disc diffusion method, eight used the automated system Vitek two compact (BioMérieux, Marcy-l'Etoile France), and two studies used both methods. For 23 studies, antimicrobial susceptibility testing was performed according to the European Committee on Antimicrobial Susceptibility Testing (EUCAST) guidelines, 20 studies used the Clinical and Laboratory Standards Institute (CLSI) guidelines, while five studies used both.

## Resistance rates, circulating genes and genetic supports in humans

Among the included studies, 49 were carried out in humans (Table 1).

The highest prevalence rate of ESBL in humans (100 %) was reported in Gabon from carriage isolates, and the lowest (2 %) in Cameroon from clinical isolates. The *CTX-M15* gene was predominant in 31 studies (31/49), followed by *TEM* (25/49), *OXA* (20/49), *SHV* (16/49), *CTX-M1* (4/49)*, CTX-M14* and *CTX-M27* (3/49 each), *CTX-M3* and *CTX-M9* (2/49 each), *CTX-M8*, *CTX-M32*, *CTX-M55*, *CTX-M127*, *act-17*, *ampR*, and *lap-2* (1/49 each).

The prevalence rate of clinical or carriage carbapenemase-producing enterobacteriaceae (CPE) ranged from 2.5–78 %. *NDM-1, NDM-4, NDM-5*, and *NDM-7* metallo-beta-lactamase genes were reported in eight studies in Cameroon, Chad, Angola, and Gabon, *OXA-48* and *OXA-181* oxacillinase genes were reported in seven studies in Gabon, Angola, Chad, Republic of Congo, and São Tomé and Príncipe. The *OXA-181* gene, a variant of *OXA-48*, was the most predominant carbapenemase gene in five studies from four countries (Angola, Chad, Republic of Congo, São-Tome and Príncipe). We did not find, in the timeframe of this study, any presence of carbapenemase coding genes in DRC and CAR studies.

Many ESBL (*bla_CTX-M_*) and carbapenemase (*bla_NDM_* and *bla_OXA_*) genes were carried by mobile genetic elements, such as transposons (Tn4651, Tn4652), class 1 and 2 integrons (Int1, Int2), non-typeable plasmids and plasmids (IncX3, IncX3, In cX4, IncFIA, IncA/C, IncFIB, IncL/M, IncN, IncY, IncFII, IncHI2, IncHIIB, IncR, CoI, CoIRNAI, IncF, IncFIB69, IncFII105, IncFII107, IncQ1). In addition to the genes for resistance to the β-lactams and to carbapenems, other genes were observed in humans, namely those for resistance to meticillin (*mecA*), aminoglycosides (*armA, rmtB*, *rmtC, aac(6')-Ib, aa [3]-IIa, ant [3], aac [3], aadA1, aadA8, aphA-3*), sulphonamides (*sul1, sul2, sul3, folA*), trimethoprim (*dhfr1, dfrA, dfrG, dfrK +G, dfrB*) tetracycline (*tetA, tetD, tetG, tetK, tetM, tetR*), quinolones (*aac(6')-Ib-cr, qnrB, qnrD, qnrS, qepA, oqxA, oqxB, crpP, gyrA, parC, parE*), fosfomycin (*fosA, ARR-3*), macrolides (*mph(A), mpbBM, msrA, erm(A), erm(B), erm(C), erm(T*)), chloramphenicol (*cat, floR, cmIA*), streptomycin (*strA, strB*), glycopeptides (*vanW*), colistin (*mcr-1*). These other resistances had prevalence rates that ranged from 0.97–96 %. The *mecA* gene was commonly associated with the chromosomal cassette SCCmecIV, SCCmecV or non-typeable SCCmec.

## Resistance rates, circulating genes and genetic supports in animals

Out of the 60 studies included, eight were in animals and carried out in Gabon (3), Cameroon (2), Angola (2), and CAR (1) (Table 2). The prevalence rates ranged from 5.9–75 % for ESBL and 50–56 % for other resistances, and were observed in gorillas, chickens, bats, pigs, cows, forest elephants, African buffaloes, chimpanzees, pigs, agile mangabeys, duikers, lowland bongos, sitatungas.

Among the β-lactamase genes, *CTX-M15* was the most predominant. *CTX-M1, CTX-M2, CTX-M14, CTX-M32, TEM, SHV, OXA, blaZ, SCO-1* were also reported.

None of the included studies revealed any carbapenemase genes but, as in humans, other genes that confer resistance to tetracycline, aminoglycosides, streptomycin, quinolones, sulphonamides, trimethoprim, chloramphenicol, methicillin, macrolides were reported.

Mobile genetic elements such as transposons, integrons (class 1 and 2) and plasmids supported the genes, except for the mecA gene, which was associated with the SCCmec Vc-type chromosomal cassette.

## Resistance rates, circulating genes and genetic supports in the environment

Among the 60 selected studies, ten revealed the presence of resistant bacteria in the environment. These studies were carried out in the Republic of Congo [2], DRC [6], Cameroon [1] and Angola [1] (Table 3). Some studies revealed the presence of carbapenemase coding genes, but none of them determined the prevalence of CPE.

ESBL-PE prevalence rate in the environment ranged from 5.2–74.42 %, and that for other types of resistance ranged from 2.8–80 %. Resistant bacteria were detected in wastewater, soil, walls, treated water for human consumption, water for animal consumption, urban sewage, domestic sewage and drinking water. The genes responsible for these resistances were β-lactamases (*CTX-M15, CTX-M1* group, *TEM, OXA, SHV, CARB, Cfx, CMY, GES, NPS, AER, VEB-3, LCR1*), carbapenemases (*AIM, IMP, VIM, NDM, OXA-48, KPC*) and all other resistance genes observed in humans and animals. The β-lactamases and carbapenemases genes were generally carried by plasmids. Only one *CTX-M15* gene, in Angola, was carried by a chromosome. As in humans and animals, the *CTX-M15* gene was the most predominant β-lactamase.

## Discussion

Antimicrobial resistance is an increasing problem for public and animal health worldwide, particularly in Africa. In Central Africa, despite the regularity of annual epidemiological reports on antibiotic resistance, there is a paucity of molecular studies, and only limited data on resistance genes are available. This absence of information on antimicrobial resistance makes it difficult to control and monitor bacterial resistance in the subregion. In fact, more knowledge of antimicrobial resistance and reasonable antibiotic use could contribute significantly to diminishing the spread of antimicrobial resistance in this region [3]. So, in order to avoid a deterioration of the health situation in the region, it is very necessary that the healthcare system adopts antimicrobial resistance surveillance strategies and implements an antibiotic use and control programme. Such strategies limit the use of antibiotics to situations where they are essential, in order to prevent antimicrobial resistance making current treatments ineffective.

Studies were carried out on several host groups (humans, animals and the environment), the most representative being studies on humans (81.67 %, 49/60) compared with those on animals (13.33 %, 8/60) and the environment (16.67 %, 10/60). Our observations corroborate the previous studies which reported also a short number of studies concerning the prevalence of ESBL-PE and CPE in West and Central Africa in animals and environment [21]. The presence of same genes in different habitats (humans, animals and environment) could suggest the potential horizontal transmission of these genes between the three domains. Although there are few studies on animals and the environment, high rates of resistance were reported, ranging from 5.9–75 % for animals, and from 5.2–74.42 % for the environment. This high burden could be explained by excessive use of antibiotics as growth stimulants and preventive treatment in animals [22], and on other hand, to anthropogenic activities that leads to antibiotic pollution of the environment [23]. In this review, the prevalence of extended-spectrum beta-lactamases (ESBL) in carriage and in infectious processes ranged from 3–100 %, and from 0.97–96 % for other resistance genes. These resistance rates are similar to those found by Ouedraogo *et al*. in West Africa, where prevalences ranged from 10–100 % for ESBLs [24]. ESBL rates found in Chad (48%) are also close to those found in East Africa (42 % in average) [25] and China (46 %) [26], but significantly higher than those found in 2012 in Germany (10–15 %) [27] or the USA (4–12 %) [28]. These differences in prevalence may be due to overuse associated with a lack of antibiotic use policy in most African and Asian countries, unlike those in the Western countries (Europe and USA). Among all the resistance genes identified, our literature review revealed high rates of isolates carrying one or more resistance genes, inducing high rates of antibiotic resistance with damaging effects on human health. Indeed, the diseases caused by these drug-resistant bacteria are lethal, due most often to the absence of effective treatments. Guillemot *et al*. established that during the past 50 years, the permanent increase in bacterial resistance has led to modifications in therapeutic recommendations [29]. For example, in response to the increased number of *Haemophilus influenzae* strains producing β-lactamase, guidelines for the treatment of otitis have favoured a combination of aminopenicillin and a β-lactamase inhibitor [30–32]. For pneumococcal meningitis, increased resistance to β-lactams has resulted in the recommendation of an injectable third-generation cephalosporin (cefotaxime or ceftriaxone) first-line prescription in combination [33] with a glycopeptide (vancomycin) [34]. In the same way, at the physician level, restricting the use of antimicrobial agents, providing locally adapted guidelines for the prudent use of antibiotics, and implementing quality control of antimicrobial therapy within a hospital, in particular within the intensive care unit, might help to minimize the selection of multidrug-resistant bacteria [35].

In the reviewed literature, among the β-lactamase genes, *bla_CTX-M15_* gene was the most predominant in humans, animals and the environment. In all the three cases it was usually carried by plasmids, which made its spread easier and therefore increased the prevalence rate of ESBL-PE in humans, animals and the environment, leading to the spread of multidrug-resistant pathogens responsible for human and animal infectious diseases [36].

There were no reports of carbapenemase genes in animals, unlike previous studies which reported *bla_KPC_, bla_OXA-48_, bla_NDM_, bla_VIM_, bla_IMP_* genes in animal samples [37]. However, carbapenemase genes were reported in humans and in the environment, highlighting pollution of the environment by human activities.

In addition to β-lactamase and carbapenemase genes, other resistance genes were reported, such as resistance genes to meticillin, aminoglycosides, quinolones, tetracycline, streptomycin, sulfonamides, trimethoprim, fosfomycin, chloramphenicol, rifampicyn, macrolides, glycopeptides, colistin, phosphonic antibiotics, streptogramins, nucleoside antibiotics, which were detected in human, animal and environmental isolates (Tables 1–3 referring to studies in humans, animals and the environment, respectively).

The included studies in this review did not allow us to identify clones.

However, the presence of the same resistance genes in all three sectors showed evidence of resistant species and clones spread between humans, animals and the environment. For example, the mecA gene, carried by the SCCmec cassette, which confers β-lactam resistance (MRSA) to *Staphylococcus aureus *, is in our review one of those distributed in the three sectors. Previous studies reported the presence of the same ST5 clone of *S. aureus* (* S. aureus* ST5) in South Africa from humans [38], in Senegal from pigs [39] and in Tunisia from the environment [40]. This means that this *S. aureus* ST5 clone was disseminated in Africa in humans, animals and the environment. Antibiotics discharged into the environment contribute to the development of antibiotic resistance in the animals that involuntarily consume them in their food. Moreover, studies have shown that the use of antibiotics in the animal sector constitutes a selection pressure and promotes the transmission of resistant mutants to humans either by direct contact or through food [41, 42]. All this proves that the struggle against antibiotic resistance can only be effective within a framework that combines human, animal and environmental health, a ‘One Health approach’.

## Conclusion

This systematic review has confirmed the scarcity of thorough studies on bacterial resistance in the Central Africa. Thus, despite the increasing prevalence rates of resistant bacteria, and the spreading of several resistance gene types in humans, animals and the environment, not enough attention is given to the monitoring of antimicrobial resistance in the subregion. This leads to a lack of data and information to efficiently tackle the increase of antimicrobial resistance. We believe that the struggle is necessarily based on the implementation of a national health policy for each country, including measures for environmental hygiene, more adequate prescription and consumption of antibiotics in human and animal health. Intersectoral collaboration (human, animal and environmental health) is essential in order to combat antibiotic resistance.

## Limitation

Annual epidemiological reporting on antibiotic resistance from most of parts of Africa is common practice. But in many countries of Central Africa, our study area, these epidemiological reports, even when they exist, do not often include data on the molecular basis of this resistance (genes). This makes it difficult to monitor antibiotic resistance.
